# The Working Classes and the Hospitals

**Published:** 1889-05-18

**Authors:** B. Burford Rawlings


### The Working Classes and the Hospitals.

By Mr. B. Burford Rawlings.

The article you publish in your issue of May 11, concerning the contributions of the working classes to the hospitals, is worthy of a careful perusal by those to whom it is especially addressed. A little plain writing about the obligation of the working classes—so-called—to the hospitals is much needed, and in this case courage is true discretion.

But I venture to think that the force of your argument is irresistible enough to have got beyond control. It is true that the working classes ought to render the hospitals substantial assistance, but it seems too much to expect that the latter can ever be supported by the voluntary, or, for that matter, the enforced, contributions of the working classes alone.

No class can be made provident as a whole, and when we are dealing with a large body of the population which at best lives from hand to mouth, and of which a large minority must subsist on incomings scarcely sufficient for the bare necessities of life in health, it is manifest that a week's serious illness will destroy the economical equilibrium, and if it involve the breadwinner may bring down the whole fabric of the home.

When a man is sick and in need of treatment he has no means to pay for, it is useless to rate him for past improvidence. Economical considerations, no less than those of humanity, demand he should be restored. But more than this, no amount of providence is sufficient to render a man working for a small daily or weekly wage, which ceases the moment he refrains, independent of the consequences of incapacitating illness. When you write that the proposed collection of £100,000 will meet but an eighth of the expenditure of the metropolitan hospitals you understate your case, because, if the working classes are to be "independent," they must be ready, as I have pointed out in a letter elsewhere, to pay for the skill of the doctors and surgeons, and for this you make no allowance, although it is the chief of the hospital wares, and would become the most costly whenever it ceases to be the gift it now is.

So long as hospitals depend upon voluntary contributions there will always be need of the help of every class, and the difficulties the institutions suffer will be enormously increased if a crusade is preached against philanthropic aid before anything is provided in its place. Should the working classes ever become independent in their time of sickness—a very remote contingency, as it seems to me—several new difficulties will demand solution—notably, where the material for teaching is to come from. It must not be forgotten that if the sick poor cannot yet see their way to do without the hospitals, the hospitals will never be able to exist independently of the sick poor. It has been said that if the outpatients ceased to come voluntarily, the hospitals would be compelled to induce them by payment. That is the bare truth put in a striking way. It is equally certain that the well-to-do classes—as classes—might do a great deal more than they do for the hospitals, and yet remain debtors to them.

